# Prevalence of Decreased Vitamin D Levels is High among Veterans with Diabetes and/or CKD

**DOI:** 10.5402/2011/109458

**Published:** 2011-07-18

**Authors:** Subhashini Yaturu, Jared Davis

**Affiliations:** ^1^Section of Endocrinology and Metabolism, Stratton VA Medical Center, 113 Holland Avenue, Albany, NY 12208, USA; ^2^Overton Brooks VA Medical Center, 511, East Stoner, Shreveport, LA 71104, USA

## Abstract

*Objective*. Vitamin D deficiency is associated with a variety of skeletal and extraskeletal problems. The aim of this study was to evaluate the prevalence of vitamin D deficiency among veterans in sunny Louisiana. *Methods*. Using the VA computerized patient record system, we searched for all 25 (OH) Vitamin D and 1, 25 (OH) vitamin D levels that were measured between 2007 and 2009. The information collected for each patient included age, body mass index, creatinine, history of diabetes and hypertension, and levels of vitamin D and PTH. We determined the number of individuals who were vitamin D insufficient and deficient. *Results*. Among 2990 studies evaluated, the mean concentration of 25 (OH) D was 22.5 ± 0.2 ng/mL, and that of 1, 25 (OH) vitamin D was 29.2 ± 0.4 ng/mL. Among them, only 695 subjects (23%) had normal values, while 889 (30%) had insufficiency, and 1405 (47%) had deficiency. Subjects with diabetes (1041) had significantly (*P* < 0.0001) lower levels (21 and 25 ng/mL) of both 25 (OH) and 1,25 (OH) vitamin D compared to subjects without diabetes (23 and 32 ng/mL). Similarly, subjects with chronic kidney disease (1128) had much lower vitamin D levels than subjects without CKD. Among subjects with diabetes, those with chronic kidney disease (512) had much lower levels of both 25 (OH) and 1,25 (OH) vitamin D than with those with normal creatinine levels. *Conclusions*. We conclude that vitamin D insufficiency and deficiency is highly prevalent in veterans, more so among subjects with diabetes and/or CKD.

## 1. Introduction

Vitamin D is an essential nutrient and is known for its role in calcium homeostasis, which is vital for optimal skeletal health. Epidemiological studies suggest a higher prevalence of metabolic syndrome and its components among individuals with vitamin D deficiency. The best marker of vitamin D status is the serum 25-hydroxyvitamin D concentration [1]. The 25-hydroxyvitamin D level should be measured in patients with suspected vitamin D deficiency. Many factors influence circulating 25-hydroxyvitamin D levels including race, season, body mass index, and age [1]. Vitamin D deficiency can cause osteomalacia, rickets, and myopathy and is associated with a variety of extraskeletal problems, including cardiovascular disease, infection, malignancy, and death [1–3]. Humans get vitamin D from exposure to sunlight, from their diets, and from dietary supplements. Calcium is actively absorbed from the small intestine in the presence of vitamin D. Vitamin D refers to vitamin D_2_ (ergocalciferol) or vitamin D_3_ (cholecalciferol). Vitamin D deficiency is defined as a serum 25-hydroxyvitamin D level of less than 20 ng/mL (50 nmol/L), and insufficiency is defined as a serum 25-hydroxyvitamin D level of 20 to 30 ng/mL (50 to 75 nmol/L). McMurtry and colleagues found that most veterans living in nursing homes suffer from vitamin D insufficiency [4]. There is limited literature concerning the prevalence of compromised vitamin D status among veterans. The aim of this study is to evaluate the prevalence of vitamin D deficiency in subjects with type 2 diabetes and/or chronic kidney disease among veterans living in sunny Louisiana.

## 2. Methods

This study is a retrospective study from Overton Brooks Veterans Administration Medical Center (VAMC) in Shreveport, Louisiana. Following approval of the protocol by the Institutional Review Board (IRB) for Ethical Research at LSUHSC and the Research and Development (R&D) Board at VAMC committees, as well as approval for the database retrieval by the regional administration (VISN), we used the database of the Veterans Health Administration (VHA) to retrieve data from the computerized patient record system (CPRS) records for patients seen between 2007 and 2008. We retrieved all 25-hydroxyvitamin D and 1,25 (OH)^2^ vitamin D levels that were measured between 2007 and 2008, along with the clinical and demographic information, and limited laboratory data, for the subjects associated with those vitamin D levels. Data collected include age, sex, body mass index (BMI), diagnosis of type 2 diabetes, hypertension, insulin use, hemoglobin A1C, calculated glomerular filtration rate (eGFR), calcium, creatinine, parathyroid hormone (PTH), and 25-hydroxyvitamin D and 1,25 (OH)^2^ vitamin D levels. The data were analyzed to compare subjects with or without diabetes. Similarly the data was compared among the groups with or without chronic kidney disease (CKD). Among subjects with diabetes, comparison was again carried out among subjects with or without CKD. 

### 2.1. Statistical Analysis

The data is expressed as mean ± standard deviation.

## 3. Results

**Figure 1:**
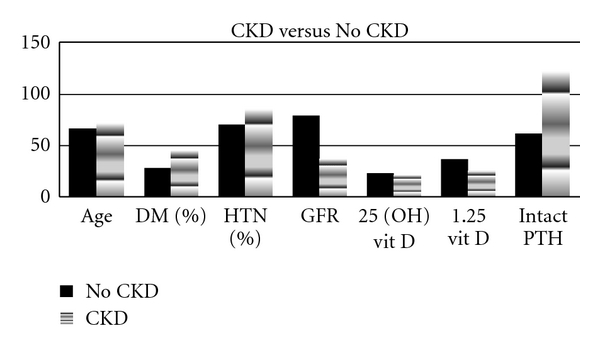
Shows the parameters in subjects with or without CKD. CKD: chronic kidney disease; DM: diabetes; HTN: hypertension; PTH: parathyroid hormone; GFR: glomerular filtration Rate.

Data were collected from 2990 subjects. Most of the study subjects included were more than 60 years old. Seventy-seven percent of subjects had 25 (OH) vitamin D levels lower than 30 ng/mL and 47% had levels lower than 20 ng/mL. Thirty-five percent (1041) of subjects had diabetes and 38% (1128) had CKD (Figure 1). Subjects with diabetes (1041) had significantly lower levels of both 25 and 1,25 (OH)² vitamin D. The clinical and laboratory data for subjects with or without diabetes are shown in Table 1. Similarly, subjects with CKD had significantly lower levels of both 25 and 1,25 (OH)² vitamin D. The data for subjects with or without CKD are shown in Table 2. Subjects with CKD were older and had a higher prevalence of hypertension compared to those without CKD. Among subjects with diabetes, subjects with CKD had markedly decreased levels of both 25 and 1,25 (OH)² vitamin D. Relative PTH levels were higher in all groups. PTH levels were elevated in relation to creatinine and declining eGFR. The comparative data of subjects with or without CKD among subjects with type 2 diabetes are shown in Table 3. The levels of 1,25 (OH)² vitamin D levels correlated positively with eGFR (*r* = 0.34; *P* < 0.0001) and negative correlation with creatinine (*r* = −  0.26; *P* < 0.0001). There was a weak positive correlation of 25 (OH) vitamin D levels to eGFR (*r* = 0.10; *P* < 0.001) and negatively with creatinine (*r* = −  0.10; *P* < 0.001), although the latter was statistically significant (Table 4).

## 4. Discussion

Vitamin D influences many physiological processes besides calcium/phosphate homeostasis, including muscle and keratinocyte differentiation, insulin secretion, blood pressure regulation, and the immune response [1, 5, 6]. Classical roles of vitamin D include the regulation of blood calcium and phosphate concentrations by actions in the intestine, bone, parathyroid, and kidney. Nonclassical roles include cell differentiation and antiproliferative actions in various cell types, including bone marrow (osteoclast precursors and lymphocytes), immune system, skin, breast, prostate epithelial cells, muscle, and intestine [5].

The data collected for the study were from male veterans residing in sunny North West Louisiana, which is rich in sunlight almost year long. It was obvious that they had been exposed to less sunlight despite the fact that they live in a region that is sunny most of the year. In general, the study population was older, with a mean age of 68 years, and most subjects had BMIs in the overweight or obese range. In this study, we used the cut-off level 75 nmol/L to define vitamin D deficiency, and we found a prevalence of 77%. Since the BMI of our patient population was generally >27 kg/m², it is unlikely that poor nutritional status caused these deficiencies. The inverse association between 25(OH) D and metabolic syndrome found in our study is consistent with that reported in the studies in the literature [7, 8]. Theoretically, it could be explained as the result of vitamin D deposition in body lipids and subsequently reduced bioavailability of vitamin D3 from skin [9]. Our study results, which show an association between vitamin D and diabetes, are similar to those from a number of published studies. The sun is the primary source of vitamin D. It is synthesized endogenously in skin to produce cholecalciferol (vitamin D3), although a small proportion (20%) of vitamin D comes from diet [10]. The marker of vitamin D status is 25-hydroxyvitamin D [25(OH)D] levels. Blood levels of 25(OH)D decline with age and obesity. It is postulated that blood levels of 25(OH)D inverse to those of diabetes reflect the prevalence of vitamin D deficiency, which increases with age and obesity [11]. Several studies have reported an association between vitamin D deficiency and diabetes. More than a decade ago, the inverse correlation of vitamin D to blood glucose levels was reported [12, 13]. The discovery of a nonclassical pathway (which is also present in renal tissue) has brought new significance to the nutritional vitamin D deficiency, given the potential role that hypovitaminosis D may play in multiple chronic diseases such as diabetes, chronic infectious processes, hypertension, cardiovascular disease, and CKD [5]. 

Our data in patients with CKD is not surprising. Despite guidelines suggesting vitamin D supplementation in CKD patients, we still noted significant vitamin D deficiencies. Patients with CKD have an exceptionally high rate of severe vitamin D deficiency that is further exacerbated by their reduced ability to convert 25-(OH) vitamin D into the active form, 1,25 dihydroxy-vitamin D. In patients with any stage of chronic kidney disease, 25-hydroxyvitamin D should be measured annually, and the level should be maintained at 30 ng per milliliter or higher, as recommended in the Kidney Disease Outcomes Quality Initiative guidelines from the National Kidney Foundation [6]. CKD patients often display vitamin D insufficiency as evidenced by insufficient circulating 25-OH-D (all stages of CKD) and progressive “circulating” 1 *α*,25-(OH)_2_D_3_ deficiency with the loss of renal 1*α*-hydroxylase (Stages 3–5 of CKD). Subjects undergoing chronic hemodialysis have been shown to have a significant survival advantage when given vitamin D therapy. Since both CKD and diabetes are associated with vitamin D deficiency, the profoundly low vitamin D levels measured in subjects with diabetes, and CKD may be secondary to an additive effect of both disease conditions. 

### 4.1. Draw Backs

As the study is a retrospective study, vitamin D supplementation details are not available.

## 5. Conclusions

The prevalence of vitamin D deficiency in the study population is quite high, especially in subjects with diabetes and/or CKD. The prevalence is higher in subjects with CKD with diabetes than in subjects with CKD without diabetes. We recommend increased sun exposure and additional vitamin D in the form of increased dietary supplementation to achieve a vitamin D sufficient state in both type 2 diabetes and CKD. 

## Figures and Tables

**Table 1:** Shows clinical and laboratory parameters comparing groups with or without diabetes. Data expressed as ±SD; DM: group with type 2 diabetes; No DM: group without type 2 diabetes; BMI: body mass index; HTN: hypertension; GFR: glomerular filtration rate; PTH: parathyroid hormone.

Diabetes versus no diabetes			
	DM	No DM	*P*-Value

Age in years	69 ± 0.34	68 ± 0.32	0.01152
BMI	31 ± 0.2	27.3 ± 0.3	1.74*E*−43
HTN (%)	88	59	2.76*E*−38
Creatinine	1.91 ± 0.05	1.52 ± 0.03	2.15*E*−09
Glucose	154 ± 2.5	106 ± 0.5	8.22*E*−63
GFR	56 ± 0.9	67 ± 0.62	1.18*E*−20
Calcium	9.2 ± 0.02	9.3 ± 0.01	0.022327
25 (OH) vit D	21 ± 0.34	23 ± 0.25	6.55*E*−08
1,25 vit D	25.5 ± 0.67	32.6 ± 0.65	8.81*E*−14
Intact PTH	105 ± 4.9	92 ± 0.36	0.032545

**Table 2:** Shows clinical and laboratory parameters in groups comparing with or without CKD. Data expressed as ±SD; DM: type 2 diabetes; CKD: chronic kidney disease; HTN: hypertension; BMI: body mass index; GFR: glomerular filtration rate; PTH: parathyroid hormone; Cre: creatinine.

CKD versus No CKD			
	No CKD	CKD	*P*-Value

Age	66.6 ± 0.31	68 ± 0.32	1.16*E*−23
BMI	28.4 ± 0.2	28.9 ± 0.2	0.052549
DM (%)	28	45	1.94*E*−20
HTN (%)	70	85	1.65*E*−23
Insulin use (%)	8	21	5.95*E*−21
Cre	1.04 ± 0.04	2.72 ± 0.07	8.7*E*−102
Glucose	119 ± 1.1	133 ± 2.25	1.11*E*−08
GFR	79 ± 0.49	36 ± 0.51	0
Calcium	9.3 ± 0.01	9.2 ± 0.01	0.000191
25 (OH) vit	23 ± 0.26	21 ± 0.33	3.77*E*−07
1,25 vit D	36 ± 0.8	24 ± 0.5	4.94*E*−30
HbA1C	6.4 ± 0.03	6.6 ± 0.05	0.003844
Intact PTH	61 ± 2.1	122 ± 4.5	2.21*E*−31

**Table 3:** Shows clinical and laboratory parameters in subjects with diabetes compared with or without chronic kidney disease. Data expressed as ± SD; DM: type 2 diabetes; CKD: chronic kidney disease; BMI: body mass index; HTN: hypertension; GFR: glomerular filtration rate; PTH: parathyroid hormone, Cre: creatinine.

Subjects with diabetes with or without CKD			
	NO CKD	CKD	*P*-Value

Age	67 ± 0.5	71 ± 0.46	0.01152
BMI	30 ± 0.3	31 ± 0.3	1.74*E*−43
HTN (%)	85	91	2.76*E*−38
Insulin Use (%)	28	46	3.6*E*−106
Cre	1.06 ± 0.01	2.8 ± 0.09	2.15*E*−09
Glucose	149 ± 2.9	157 ± 4	8.22*E*−63
GFR	77 ± 0.9	35 ± 0.73	1.18*E*−20
Calcium	9.3 ± 0.02	9.2 ± 0.02	0.022327
25 (OH) vit	22.3 ± 0.47	19.6 ± 0.49	6.55*E*−08
1,25 vit D	31.9 ± 1.2	22.9 ± 0.75	8.81*E*−14
HbA1C	6.9 ± 0.05	6.8 ± 0.06	2.65*E*−89
Intact PTH	63 ± 0.3	124 ± 0.6	0.032545

**Table 4:** Shows clinical and laboratory parameters in subjects with normal creatinine with or without DM. Data expressed as ±SD; DM: type 2 diabetes; BMI: body mass index; HTN: hypertension; GFR: glomerular filtration rate; PTH: parathyroid hormone; Cre: creatinine.

	No DM	DM	*P* value
Age	67 ± 0.4	68 ± 0.4	<.0001
BMI	27 ± 0.17	31 ± 0.27	<.0001
HTN (%)	67	86	<.0001
Cre	1.05	1.1	<.001
Glucose	105	149	<.0001
GFR	78 ± 0.56	74 ± 0.95	<.0001
Calcium	9.3	9.2	NS
25 (OH) vitamin D	23.7 ± 0.30	22.3 ± 0.45	<.0001
1,25 vit D	37.4 ± 0.94	31.9 ± 1.17	<.0001
Intact PTH	61.7 ± 2.4	65.1 ± 3.5	NS
